# The male biological clock is ticking: a review of the literature

**DOI:** 10.1590/S1516-31802008000300012

**Published:** 2008-05-01

**Authors:** Fabio Firmbach Pasqualotto, Edson Borges Júnior, Eleonora Bedin Pasqualotto

**Keywords:** Male, Erectile dysfunction, Infertility, Diabetes complications, Heart, Masculino, Disfunção erétil, Infertilidade, Complicações do diabetes, Coração

## Abstract

The term biological clock is usually used by physicians and psychologists to refer to the declining fertility, increasing risk of fetal birth defects and alterations to hormone levels experienced by women as they age. Female fecundity declines slowly after the age of 30 years and more rapidly after 40 and is considered the main limiting factor in treating infertility. However, there are several scientific reports, chapters in books and review articles suggesting that men may also have a biological clock. The aim of our study was to conduct a review of the literature, based on the Medical Literature Analysis and Retrieval System Online (Medline), to evaluate the male biological clock. After adjustments for other factors, the data demonstrate that the likelihood that a fertile couple will take more than 12 months to conceive nearly doubles from 8% when the man is < 25 years old to 15% when he is > 35 years old. Thus, paternal age is a further factor to be taken into account when deciding on the prognosis for infertile couples. Also, increasing male age is associated with a significant decline in fertility (five times longer to achieve pregnancy at the age of 45 years). Patients and their physicians therefore need to understand the effects of the male biological clock on sexual and reproductive health, in that it leads to erectile dysfunction and male infertility, as well as its potential implications for important medical conditions such as diabetes and cardiovascular diseases.

## RESUMO

O termo relógio biológico é geralmente usado por médicos e psicólogos para se referir ao declínio da fertilidade, aumento no risco de defeitos congênitos, e níveis hormonais alterados que a mulher com o passar dos anos apresenta. A fecundidade feminina declina paulatinamente após os 30 anos e mais rapidamente após os 40 anos e é considerada como o principal fator limitante nos tratamentos da infertilidade. Entretanto, existem vários artigos científicos capítulos de livros e artigos de revisão sugerindo que o homem também apresente um relógio biológico. O objetivo do nosso estudo foi realizar uma revisão no Medline (Medical Literature Analysis and Retrieval System Online) para avaliar o relógio biológico masculino. Após ajustar para outros fatores, foi demonstrado que a probabilidade de um casal apresentar uma demora superior a 12 meses para engravidar praticamente duplica de 8% quando o homem possui idade inferior a 25 para 15% quando possui idade superior a 35 anos; desta forma, a idade paterna é um fator a ser levado em consideração quando se decidir sobre o prognóstico de um casal infértil. Além disso, a idade avançada está associada com um declínio significante na fertilidade (tempo para engravidar superior a cinco vezes aos 45 anos de idade), sendo independente de idade da parceira, freqüência de relação sexual e estilo de vida, assim como os efeitos de outros fatores de risco para subfertilidade. Os pacientes e seus médicos devem entender os efeitos do relógio biológico na saúde sexual e reprodutiva, levado à disfunção erétil e infertilidade, assim como às potenciais contribuições para condições médicas como diabetes e doenças cardiovasculares.

## AGING AND MALE INFERTILITY

It is well known that practically no children are born to mothers aged over 50 years and all older recent fathers and fathers-to-be share the characteristic that they have younger partners.^1^ The discrepancy in the reproductive arena between males and females is astonishing, and the reduced fertility and higher reproductive risks associated with advancing maternal age raise the question of whether advanced paternal age might also be associated with compromised fertility and increasing risks.^2-4^

In contrast to female reproductive functions, male functions do not cease abruptly, and androgen production and spermatogenesis continue throughout life.^5,6^ However, evaluating the possible decline in semen quality is somewhat difficult. Some men are reluctant to provide semen samples unless actively concerned about their fertility. For instance, 20% of the individuals in population-based studies are typically recruited among young men willing to provide semen samples.^7^ thus constituting an inevitable participation bias in such studies.^8,9^ In addition, mostly of the published studies on sperm output among older men are largely restricted to patients attending infertility clinics, where few are more than 50 years old.^10^ An undefined, but probably high proportion of such men have unrecognized defects in sperm production and/or function. Furthermore, access to such specialized medical services may be strongly influenced by non-biological factors, and the results from infertility clinics cannot be reliably extrapolated to the general male population.

## AIM AND METHOD

The aim of our study was to conduct a review of the literature, based on the Medical Literature Analysis and Retrieval System Online (Medline), to evaluate the male biological clock. In our search in the Medline database, the following words were used in combination with the words male and age, such that for “infertility”, 2,576 articles were retrieved; “erectile dysfunction”, 2,129 articles; “heart disease”, 62,638 articles; “diabetes”, 38,946 articles; and “metabolic syndrome”, 2,810 articles. We only ordered and used the papers involving the study subject that were written in English.

## FINDINGS

The effects of paternal age on a couple’s fertility are real and may be greater than has previously been thought. To explain the age-dependent changes observed in semen quality, two issues should be considered.^10-13^ First, cellular or physiological changes due to aging have been described in the testicles, seminal vesicles, prostate and epididymis. Age-related narrowing and sclerosis of the testicular tubular lumen, decreased spermatogenic activity, increased germ cell degeneration and decreased numbers and function of Leydig cells have been found in autopsies on men who died from accidental causes.^14^ Smooth muscle atrophy and decreased protein and water content, which occur in the prostate with aging, may contribute towards decreased semen volume and sperm motility. Furthermore, the epididymis, which is hormonally sensitive tissue, may undergo age-related changes. Hormonal or epididymal senescence may lead to decreased motility in older men. Secondly, increasing age implies more frequent exposure to exogenous damage or disease.^10^ In addition to age per se, factors such as urogenital infections, vascular diseases or accumulation of toxic substances (cigarettes) may be responsible for worsening semen parameters. Indeed, a retrospective cross-sectional study among 3,698 infertile men showed an accessory gland infection rate of 6.1% in patients aged < 25 years but 13.6% in patients > 40 years, and total sperm counts were significantly lower in patients with accessory gland infection.^11^ In addition, age-dependent increases in polychlorinated biphenyls (PCBs) in men have been described, while in men with normal semen parameters, the PCB concentration is inversely correlated with sperm count and progressive motility.^15^ The cadmium concentration in the human testis, epididymis and prostate also increases with age, although lead and selenium concentrations in reproductive organs remain constant over the whole age range.^16,17^

Handelsman and Staraj demonstrated that, after excluding men with different diseases associated with diminished testicle size, the specific effects of age on testicular volume appeared only in the eighth decade of life.^18^ Among healthy men in this age group, the testis volume is 31% lower than among 18 to 40-year-old men.^19^ However, a recent study showed a decline in testicular volume over time, especially after the age of 45 years.^5^ The morphological characteristics of aging testes include reduced numbers of Sertoli cells and accumulation of cytoplasmic lipid droplets in these cells, and reduced numbers of Leydig cells, which may also be multinucleated.^20-22^ Tubule involution is associated with enlargement of the tunica propria, thus leading to progressive sclerosis in parallel with reduction of the seminiferous epithelium, with complete tubular sclerosis as an endpoint.^23^ Testicular sclerosis is associated with defective vascularization of the testicular parenchyma and with systemic arteriosclerosis in affected men.^24^ The arteriographic patterns of the epididymis and testes support these findings and are correlated with the degree of systemic arteriosclerosis.^24^ In addition, age-dependent alterations of the prostate are well known and, while they are detectable histologically in 50% of 50-year-old men, 90% of men aged over 90 years present such alterations.^25^

It has been demonstrated that men over 35 years old are twice as likely to be infertile (defined as the inability to initiate a pregnancy within 12 months) as are men under 25 years old.^4^ Among couples undergoing fertility treatment consisting of intrauterine insemination, the amount of time needed to achieve pregnancy increases significantly with the age of the male member of the couple. Furthermore, after controlling for maternal age, couples in which the man is over 35 years old have a 50% lower pregnancy rate than do couples in which the man is not more than 30 years old.^26^

## FERTILITY OF AGING MEN

Without any type of doubt, male fertility is basically maintained until very late in life, and it has been documented scientifically to continue until an age of more than 90 years.^27^ In addition to female age, further confounders such as reduced coital frequency, increased incidence of erectile dysfunction and smoking habits have to be considered in studies analyzing male fertility.^28^ All studies that have focused on non-clinical populations have found a significant negative relationship between male age and couples’ fertility.

A retrospective study on a large sample of European couples that analyzed the risk of difficulties (due to adverse pregnancy outcomes such as ectopic pregnancy, miscarriage or stillbirth, or due to delayed conception) and the risk of delay in pregnancy onset, found that it is higher if both partners are advanced in age.^29^ Age-related changes were also found in a prospective study that estimated day-specific likelihoods of pregnancy in relation to ovulation.^30^ The frequency of sexual intercourse was monitored by means of keeping a diary, and ovulation was monitored according to basal body temperature measurements. According to one study, fertility for men aged over 35 years is significantly reduced, and the age effect on men aged 35-40 years is about the same as when the intercourse frequency drops from twice a week to once a week.^31^ In studies dealing with subfertile couples, significant decreases in pregnancy rates and increases in the time taken to become pregnant were observed with female aging but not with male aging, thus possibly indicating that male age-dependent alterations are masked by the infertility as such.^32^

Through assisted reproduction methods, the prerequisites for natural conception, such as motility or fertilizing capacity, are circumvented. In fact, the more invasive the treatment is, the less important male age appears to be. Therefore, the success rates of intracytoplasmic sperm injection (ICSI) or in vitro fertilization (IVF) are not associated with male age.^33-36^ On the other hand, the success rate of intrauterine insemination (IUI), which is a method requiring much higher sperm quality and capability, is without question related to male age.^26,37^

## GENETIC RISKS OF AGING MALES

Women should no longer be viewed as solely responsible for age-related fertility and genetic problems. Infertility is not just a woman’s problem, and awareness of the effects of the male biological clock will allow couples and their physicians to proceed with proper testing, diagnosis and (if needed) treatment for the male partner.^38^ In men, advancing age decreases semen volume, percentage of normal sperm and sperm motility. While these factors adversely affect fertility, the genetic integrity of sperm is at risk.^39^

Although the association between advancing maternal age and increased incidence of birth defects has long been recognized, paternal age has been considered to be less relevant. Recent data suggest that paternal age does matter, and that the genetic quality of sperm does decline with age.^40^ In fact, for instance, the rate of genetic abnormalities that occur during spermatogenesis increases.^41-43^ Moreover, the frequency of numerical and structural abnormalities in sperm chromosomes increases with aging.^41-43^ This age-related increase in sperm cells with highly damaged DNA results from both increased DNA double-strand breaks and decreased apoptosis during spermatogenesis.^42,43^ Reichenberg et al. recently reported a significant association between advancing paternal age and the risk of autism spectrum disorder (ASD) in their children.^44^ The offspring of men aged 40 years or over were 5.75 times more likely to have ASD than were the offspring of men younger than 30 years, after controlling for year of birth, socioeconomic status and maternal age. Also, older men were at higher risk of fathering a child with schizophrenia.^45^ Men over 40 years old were more than twice as likely to have a child with schizophrenia as were men in their twenties.^45^ A similar influence of paternal age on the risk of having a child with Down’s syndrome has also been reported, and paternal age was a factor in half of the cases of Down’s syndrome when the maternal age exceeded 35 years.^46^ Other investigators found that the rate of miscarriages increased with advancing paternal age when the maternal age was more than 35 years.^30^ Thus, there is convincing evidence to suggest that there is an effect from paternal age alone as well as a combined effect from advancing paternal and maternal age, relating to increased risks of genetic abnormalities leading to miscarriage or disease in their children.

### Declining testosterone levels

Similarly to women, aging in men is also associated with declines in sex hormone levels. The decrease in hormone levels in men is not as steep or as sudden as seen in women during the menopause, but its effects can be significant.^6^ Blood testosterone levels decline with age in most men, even in those who are healthy, and this decline probably begins around the age of thirty.^47^ The rate of decline varies greatly between individuals and most of the data are to be found in studies on Caucasian men. Both cross-sectional and longitudinal studies confirm this decline.^48-51^ Decreased production of testosterone by the testes is the main reason for lower testosterone levels in older men.^51^ From a clinical standpoint, the pertinent question is whether this decline in testosterone is significant enough for many older men to consider testosterone replacement therapy.

The decline of around 1% per year in testosterone levels after the age of 30 years has been termed the *andropause.*^52^ However, a more technically accurate description for the decline in testosterone might be “symptomatic hypogonadism in aging males.” Hypogonadism is not defined by any specific level of serum androgens because the testosterone level that causes dysfunction varies widely among individuals.^53^ The symptoms associated with symptomatic hypogonadism in aging males include decreased libido, decreased muscle mass, decreased bone mineral density, increased fat mass, central obesity, insulin resistance, emotional irritability, dysphoria and erectile dysfunction. In a recent article in which low serum testosterone was defined as a total testosterone level of less than 250 ng/dl (8.68 nmol/l), or a free testosterone level of less than 0.75 ng/dl (0.03 nmol/l), men with low testosterone levels had a mortality rate of 35% over the eight-year study period, compared with a mortality rate of only 20% among men with normal testosterone levels. This trend persisted even after controlling for other relevant variables in the study population.^54^

The use of testosterone in older men has become of increasing interest to both the medical and lay communities over the past decade. Even though knowledge of the potential benefits and risks of male assisted reproductive technology (ART) has increased dramatically, there is still much that needs to be determined.^6,47^ Although ART has resulted in increased bone mineral density, the impact on fracture risk is unknown. ART can lead to an increase in muscle mass and a decline in fat mass, but whether this translates into significant clinical changes in strength or function has not yet been proven.^47^ Indiscriminate use of testosterone supplements can increase the risks of prostate hyperplasia (and perhaps cancer), coagulation disorders (resulting in cerebrovascular damage), dyslipidemia, and male infertility.^6,47,51^ Simply replacing testosterone is an inadequate and ineffective way to treat chronic progressive conditions with multiple causal factors (such as smoking).

On the other hand, data showing that ART, with appropriate patient screening and monitoring, is relatively safe in the short term continue to accumulate, thereby indicating that many of these early possible adverse effects can be managed. In fact, a recent report has suggested that the risks to men undergoing controlled testosterone replacement therapy for laboratory-confirmed hypogonadism may be lower than previously believed.^55^ Well-controlled large multicenter trials need to be undertaken in order to ascertain whether or not ART increases the risk of developing or worsening cardiovascular or prostate disease.

## MEDICAL CONDITIONS

A link between aging and diabetes seems to exist. Both type 2 diabetes and metabolic syndrome (which involves both pre-diabetes symptoms and cardiovascular risk factors) are strongly associated with low testosterone levels.^56^ Grundy found that 40% of men with type 2 diabetes between the ages of 40 and 49 years were hypogonadal, and the rate was nearly 55% among men in their seventies.^57^ Interestingly, the low testosterone levels found in men with type 2 diabetes may be related to the correspondingly high prevalence of erectile dysfunction (ED) among men with diabetes. The latter is estimated to range from 35% to 75%.^57,58^ Several published studies have now shown that ED is associated with depression, benign prostatic hyperplasia (BPH) and cardiovascular disease (CVD). However, ED is also associated with age-related biological changes, and aging is associated with several chronic conditions and diseases. For instance, the prevalence of ED is significantly higher among patients undergoing treatments for heart disease and hypertension.^59^ Treatments for hypertension may contribute towards ED, which may help explain the increased incidence of ED in such patients. However, the reverse relationship may be true; ED may be considered to be a hypertension marker.^59^

Depression may both contribute towards and link ED and CVD. Erectile dysfunction is associated with well-established negative psychological effects, primarily depression and anxiety.^60^ Patients with ED are more likely to become depressed than are those without ED, and therefore may be at increased risk of developing CVD. An association between ED and BPH has been described.^61^ Erectile dysfunction, CVD, depression and BPH are all common conditions among older men and, because these conditions appear to be strongly correlated, a multidisciplinary approach to future research and clinical practice is warranted.

## CONCLUSIONS

Aging among males leads to several clinical consequences of utmost importance. Better understanding of the male biological clock may reduce the adverse outcomes in the offspring of older fathers and may help facilitate the progress towards reducing the risks of diabetes, BPH, erectile dysfunction, depression and cardiovascular disease.
